# dPORE-miRNA: Polymorphic Regulation of MicroRNA Genes

**DOI:** 10.1371/journal.pone.0016657

**Published:** 2011-02-04

**Authors:** Sebastian Schmeier, Ulf Schaefer, Cameron R. MacPherson, Vladimir B. Bajic

**Affiliations:** Computational Bioscience Research Center (CBRC), King Abdullah University of Science and Technology (KAUST), Jeddah, Saudi Arabia; The Centre for Research and Technology, Greece

## Abstract

**Background:**

MicroRNAs (miRNAs) are short non-coding RNA molecules that act as post-transcriptional regulators and affect the regulation of protein-coding genes. Mostly transcribed by PolII, miRNA genes are regulated at the transcriptional level similarly to protein-coding genes. In this study we focus on human miRNAs. These miRNAs are involved in a variety of pathways and can affect many diseases. Our interest is on possible deregulation of the transcription initiation of the miRNA encoding genes, which is facilitated by variations in the genomic sequence of transcriptional control regions (promoters).

**Methodology:**

Our aim is to provide an online resource to facilitate the investigation of the potential effects of single nucleotide polymorphisms (SNPs) on miRNA gene regulation. We analyzed SNPs overlapped with predicted transcription factor binding sites (TFBSs) in promoters of miRNA genes. We also accounted for the creation of novel TFBSs due to polymorphisms not present in the reference genome. The resulting changes in the original TFBSs and potential creation of new TFBSs were incorporated into the Dragon Database of Polymorphic Regulation of miRNA genes (dPORE-miRNA).

**Conclusions:**

The dPORE-miRNA database enables researchers to explore potential effects of SNPs on the regulation of miRNAs. dPORE-miRNA can be interrogated with regards to: a/miRNAs (their targets, or involvement in diseases, or biological pathways), b/SNPs, or c/transcription factors. dPORE-miRNA can be accessed at http://cbrc.kaust.edu.sa/dpore and http://apps.sanbi.ac.za/dpore/. Its use is free for academic and non-profit users.

## Introduction

MicroRNAs (miRNAs) are ∼22 nucleotides long non-coding RNAs that in mammals predominantly act as post-transcriptional regulators and affect gene regulation by decreasing transcript levels mainly through the degradation of mRNA [1], [2]. Canonical miRNA biogenesis begins with the transcription of pri-miRNAs by RNA polymerase II [3], [4], [5], which suggests that miRNA genes are controlled through similar regulatory mechanisms as protein-coding genes. Some new evidence shows that some pri-miRNAs are also transcribed by RNA polymerase III [6], [7]. Cleaving of pri-miRNAs through microprocessor complex Drosha (RNase II endonuclease) and DGCR8 (a double-stranded RNA binding protein) results in forming 60∼70 nt pre-miRNAs [8], [9]. These are exported into the cytoplasm by Exportin-5 and its co-factor RanGTP [10] and finally cleaved by Dicer, an RNase III endonuclease, which leads to mature miRNAs after strand separation [2], [11].

The effect of miRNAs on mRNA has recently been the focus of several computational studies, e.g. a number of tools have been developed for predicting miRNA targets (see [12] for a review). On the other hand, the transcriptional machinery that controls the transcription of miRNA genes is currently not well understood. Recent efforts to elucidate what causes miRNAs to be transcribed include experimental and computational methods to elucidate the regulatory regions or transcription start sites (TSSs) of the miRNA genes [13], [14], [15], [16], [17] and the effect of transcription factors (TFs) on miRNA gene transcription [18]. miRNAs can be generated from their own transcriptional units (intergenic), or from within a protein-coding host gene (intragenic) [2]. Several miRNAs can be transcribed together as a single pri-miRNA [19], [20], [21] and are thus transcriptionally co-regulated. Reciprocally, a mature miRNA can stem from several locations in the genome, potentially under the control of several promoter regions [2].

Single nucleotide polymorphisms (SNPs) within a DNA sequence are point variations whose least abundant allele is present in at least 1% of the human population [22], [23] and occur throughout the genome. SNPs found in the human population have been linked to the development of diseases and the response of patients to drugs [24], [25], [26]. Those SNPs that appear in the coding regions may or may not change the polypeptide sequence of the encoded protein, so-called synonymous or non-synonymous SNPs, respectively [27]. Also, SNPs can occur in the regulatory and intergenic regions on the DNA. These may affect gene splicing or TF binding [24], [27] and deregulate transcriptional response.

Previously compiled resources related to both SNPs and miRNAs, like the microRNA Target Site (PolymiRTS) database [28] and dbSMR [29], investigate how SNPs affect the binding of miRNAs to their target protein-coding mRNAs and in this manner influence and regulate the translation of the mRNAs (down-stream effects).

Our Dragon Database of Polymorphic Regulation of miRNA genes (dPORE-miRNA) complements the previous studies. We studied promoter regions of human miRNA genes (intergenic as well as intragenic) for the potential effects that SNPs may have due to their overlap with putative TF binding sites (TFBSs) or as a potential cause for the creation of novel TFBSs on positions where no TFBS was mapped. We compiled miRNA gene promoters from several sources and searched for TFBSs that map to them. The identified TFBSs have been overlaid with known SNPs to provide a basis for a comprehensive overview of how the transcriptional machinery that regulates miRNA genes might be affected by SNPs. Work in this direction has previously been done for protein-coding genes [30], [31].

A recent database, miRGen 2.0 [32] focuses on the regulation of miRNAs. For each miRNA, miRGen 2.0 extracts the content of the miRNA promoter in the form of predicted TFBSs. In miRGen 2.0 an SNP is only indicated if it is overlapping with a predicted TFBS. However, no additional information about the potential effects of these SNPs is available in miRGen 2.0. Furthermore, no search interface is provided to search specifically for TFBSs that are influenced by SNPs.

In contrast, our approach elucidates what influences SNPs potentially have on the regulatory machinery via modifying TFBSs or creating new TFBSs as well as the consequences of such changes. The presence of an SNP on a binding site can result in several different effects. Explicitly, an SNP can lead to (1) a loss of the binding site, (2) a change of binding site, meaning that due to the SNP a different TF binds the region or that the same TFs can bind to the site but with a different affinity, (3) a creation of a new binding site in a region where previously no binding site was known, or (4) no change at all.

The dPORE-miRNA database incorporates the information mentioned above. In order to make our database of interest to a wide variety of users, we incorporated miRNA-disease [33], miRNA-target [34], and miRNA-pathway associations. Thus, the starting point for a study with dPORE-miRNA can be a specific miRNA, miRNA target, disease, or biological pathway. Alternatively, data may be queried from the perspective of a specific SNP or TF of interest. In summary, the database provides a user-friendly web interface for the exploration of specific miRNAs, the SNPs that may have influence over transcription initiation and regulation of miRNA genes, and the affected TFBSs in miRNA promoters. To the best of our knowledge, a database resource that provides such a set of information and features does not yet exist.

## Methods

All promoter regions are compiled from the human genome build HG18. MiRNA promoter regions have been extracted based on information from [13], [14], [15], [16], [17] and the UCSC Genome browser [35]. We used two methods of promoter extraction. The first method relies on sequence-based promoter regions that have been extracted as they were defined in [14], [15], [17]. The second method uses TSS-based promoter regions, which have been extracted upstream from the TSS positions and cover segments of 5000 bp. Information about the TSS locations has been derived from [13], [16], [35]. Where genomic positions were given in HG17, we have converted them to HG18 using the UCSC liftover program [35] (see Table 1 for detailed numbers of promoters and miRNAs).

**Table 1:** Promoter sets.

Promoter set	Method	MiRNA	Promoter	Avg. promoter size
Marson et al.	Sequence	297	360	677.8±1,055.2
Ozsolak et al. (MALME)	TSS	146	108	5,000.0±0.0
Ozsolak et al. (MCF7)	TSS	137	100	5,000.0±0.0
Ozsolak et al. (UACC62)	TSS	146	107	5,000.0±0.0
Zhou et al.	Sequence	99	99	347.9±5.8
Fujita et al.	Sequence	79	59	570.0±516.3
Corcoran et al.	TSS	65	40	5,000.0±0.0
UCSC	TSS	705	718	5,078.0±203.7

Table 1 shows the number of miRNAs and promoters gathered from different sources. We also show the method of promoter extraction.

All SNP data has been extracted from the UCSC Table browser according to dbSNP130 [36] from the HG18 track. In total we gathered 18,833,531 SNPs from the resource. From our promoter data (Table 1), we found that 22,315 SNPs could be mapped to miRNA promoter regions. The distribution of various dbSNP classes of the SNPs is shown in Table 2. The number of SNPs in each group of promoter regions is shown in Table 3.

**Table 2:** SNP class distribution of the 22,315 miRNA promoter SNPs.

dbSNP class	# SNP	% of amount of SNPs
single	17,475	74.40
deletion	1,913	8.14
insertion	3,298	14.04
mixed	111	0.47
in-del	551	2.35
mnp	80	0.34
named	55	0.23
microsatellite	5	0.02

The table shows for each of dbSNP class of SNPs the number and percentage of SNPs that have been found in the promoter regions. The first three SNP classes together make over 96% of all SNPs.

**Table 3:** Number of SNPs per promoter set.

Promoter set	# SNPs
Marson et al.	1,182
Ozsolak et al. (MALME )	2,992
Ozsolak et al. (MCF7)	2,766
Ozsolak et al. (UACC62)	2,925
Zhou et al.	188
Fujita et al.	140
Corcoran et al.	1,157
UCSC	18,312

The table shows the number of SNPs in each individual promoter set.

With the BIOBASE MATCH™ program version 8.3 [37], we mapped TFBSs to the individual promoter regions (Table 1) using 220 non-redundant vertebrate BIOBASE TFBS matrix models (BIOBASE Knowledge Library, including TRANSFAC [38]). The vertebrate minimum false positives motif profiles were used. Table 4 shows an overview of the numbers of identified TFBSs and unique motifs (out of 220) in the individual promoter sets.

**Table 4:** Overview of TFBS predictions for promoter sets.

Promoter set	Total TFBS predictions	Unique TFBS motifs	TFBSs overlapping SNPs
Marson et al.	13,058	201	780
Ozsolak et al. (MALME )	27,103	207	1,660
Ozsolak et al. (MCF7)	25,448	207	1,556
Ozsolak et al. (UACC62)	26,990	207	1,615
Zhou et al.	1,690	165	119
Fujita et al.	1,693	170	101
Corcoran et al.	9,779	199	644
UCSC	178,214	217	12,329

The table shows the number of TFBS predictions for each promoter set, the number of unique motifs found in each individual promoter set, and the number of TFBS predictions that are overlapping with a SNP.

All SNPs that appear in the promoter regions of miRNA genes have been studied (see Table 4). From these, all SNP effects on TFBSs have been investigated and each pair of SNP-TFBS has been examined in detail as follows:

1. A region from the promoter comprising of 30 nucleotides upstream and 30 nucleotides downstream around the SNP position was extracted, including the SNP.
2. Within this region each observed variation of the SNP has been considered and the so modified region searched again for TFBSs with the same method as described above.
3. The potential SNP effects were recorded for each variation in the form of prediction scores, matching binding motifs, and the loss or gain of binding motifs.

In addition, all SNPs that appear in the promoter region but do not overlap with a TFBS have been examined if they cause the creation of new predicted TFBSs. These newly predicted TFBSs could only be found due to the observed SNP variation. In this way, we catalogued the potential effects of SNPs on TFBSs present within miRNA promoters, as well as those TFBSs introduced by polymorphisms. In this way, one has the possibility to explore four different effects that SNPs may cause (see Table 5).

**Table 5:** The effect of the SNPs.

Effect	Description
unmodified	The introduced change in the DNA sequence due to the observed state of the SNP has no effect on the predicted TFBS; all scores for the TFBS are the same.
modified	The introduced change in the DNA sequence due to the observed state of the SNP causes a modification in prediction strength (“matrixscore” and “corescore”) of the predicted TFBS
loss	The introduced change in the DNA sequence due to the observed state of the SNP causes the loss of the TFBS.
new	The introduced change in the DNA sequence due to the observed state of the SNP makes the prediction of a new TFBS possible.

The table shows the four effects that a SNP can have on the regulation of a miRNA gene.

Finally, we incorporated important associations between miRNAs and information intended to aid in the interpretation of search results: a/miRNA-to-disease associations from the PhenomiR database [33]; b/miRNA-to-target associations, experimentally verified from the Tarbase database [34]; c/target-to-biological pathway associations from KEGG pathways [39]. In this way we indirectly map associations between miRNAs and biological pathways and enable searching via KEGG pathways in dPORE-miRNA.

## Results

**Figure 1:**
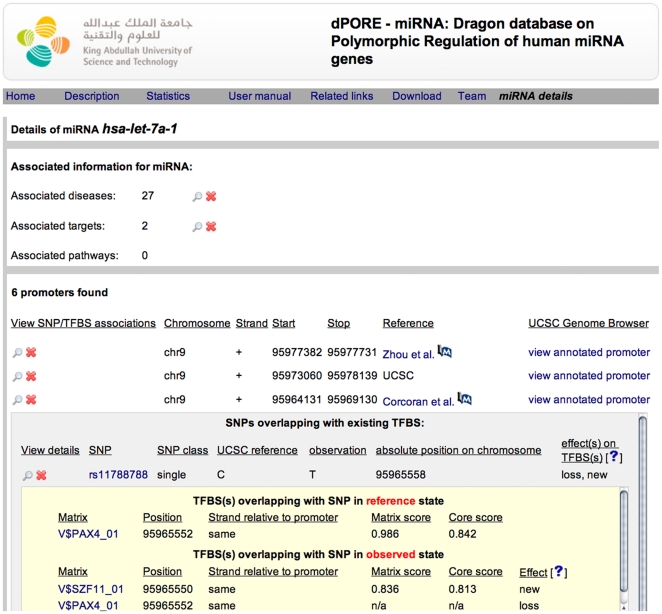
Result page example. The figure shows the result page for miRNA hsa-let-7a-1. It shows the expanded detailed view for SNP rs11788788 found on chromosome 9 in the promoter extracted from Corcoran et al. [13]. In this case the observed state of the SNP leads to a loss of the overlapping TFBS (V$PAX4_01) and the creation of a new binding site (V$SZF11_01).

Information regarding promoters, miRNAs, SNPs, TFBSs, and the effects of SNPs on TFBSs has been incorporated into a relational MySQL database (version 5.1). To provide the best possible uptime for the database and the web-interface we installed the database at two locations: http://cbrc.kaust.edu.sa/dpore and http://apps.sanbi.ac.za/dpore/. The web-interface allows for searching the database for specific miRNA identifier, miRNAs that are associated to a specific disease, miRNAs that are associated to a specific pathway, or miRNAs that are known to target a mRNA of a specific protein. Regardless of the starting point of the user query, be it miRNA, disease, target, or pathway, a list of miRNAs related to the user query is given. After the miRNA of interest has been selected, the result page summarizes information regarding the miRNA by displaying all disease, pathway and target associations. Additionally, miRNA promoter data is displayed according to the promoter's source (see Table 1). The promoters are linked to the UCSC Genome browser, which includes custom tracks uploaded by dPORE-miRNA. The custom tracks display all SNPs overlapping putative TFBSs. Viewing each promoter in detail on the result-page displays for each SNP the potential effects on the transcriptional regulation of the miRNA gene. An extended view lists all SNPs overlapping binding motifs and the possible effects (see Table 5) on the TFBS due to the SNP. Information about the binding motif, its location and TFs that potentially bind the motif, are also available (see Figure 1). For users requiring information on specific SNPs or TFs, dPORE-miRNA offers the possibility to search according to specific SNP or TF identifiers. In this manner it is possible to decipher what effects a specific SNP may have on the regulation of miRNA genes, or, which miRNA promoters contain a predicted TFBS for a specific TF that is affected by the SNP.

A comprehensive user manual is available on the website and provides an easy walkthrough of dPORE-miRNA.

## Discussion

SNPs are known to be associated with a multitude of diseases and, specifically, it has been shown that SNPs in the regulatory regions can affect the binding of TFs or even lead to the complete loss of function of a particular binding site [24]. Similar efforts to catalogue SNPs that might influence the transcription regulation of protein-coding genes led to the compilation of SNP@Promoter [31] and PupaSuite [30] databases.

There is a limited understanding of the regulation of miRNAs. Even less is known about how SNPs may influence this regulation and what consequences such an influence may have on diseases. miRNAs are known to play a role in human diseases through their effect on gene regulation [40], [41], [42], [43]. Approaches implemented in PolyMIRTS [28], and dbSMR [29] try to answer the question on how SNPs affect miRNA binding to miRNA targets. The database miRGen 2.0 [32], on the other hand, shows putative TFBSs in miRNA promoter regions and the overlap of these with SNPs but comes short of providing any information on the consequences that such overlaps may cause. In addition, it is not possible to search specifically for TFBSs that overlap with an SNP. Our dPORE-miRNA database provides answers to the question: ‘What are the potential consequences of the presence of SNPs in the promoter regions of miRNA genes?’ and extends these further to pathways and diseases that considered SNPs may influence. In this way dPORE-miRNA should be of interest to a broad range of users.

dPORE-miRNA contains information on the possible differential regulation of miRNAs in human sub-populations. Moreover, it stores a total of 734 different miRNA-targets. Among these are the products of 30 genes that are known to be implicated in various cancers through missense mutations according to the Sanger Cancer Gene Census [44]. The effect of TF binding on the expression levels of miRNAs has been experimentally shown [45]; on the other hand, there are cases where changes in the promoter composition through SNPs lead to TFBS losses and with that to modified expression levels of the controlled transcriptional unit [24], [27]. By utilizing dPORE-miRNA to extract information about: a/the SNPs that reside in the regulatory regions of miRNAs and b/the potential effects that these SNPs may have on TF binding, the user can gain an insight into potential causes for changes in miRNA expression levels that might aid to explain the differential expression of known oncogenes in cancerous tissue. For example, EGFR is a well-studied cancer gene that is differentially expressed in a number of cancers and is used in clinical settings as a therapeutic target [46], [47], [48]. Human EGFR is targeted by the products of four human miRNA genes (among them hsa-mir-16-2). This miRNA is shown to be ubiquitously expressed among human and rat tissues, and at concentrations greater than any other miRNA [49]. dPORE-miRNA allows for the detailed exploration of SNP-influenced TF binding in the regulatory regions of these miRNA genes, which could unlock valuable information on the expression levels of this important cancer gene. It is know that hsa-mir-16-2 is regulated by STAT5 [50]. dPORE-miRNA documents the loss of a binding site for TF STAT5 in the upstream region of hsa-mir-16-2 through SNP rs60640467, which could have potential consequences for the transcription initiation of the hsa-mir-16-2 gene. This could have effects on the expression of the miRNA target, EGFR, and thus may exert its influence in cancers. This illustrates a possibility to use dPORE-miRNA for the detailed exploration of SNP-influenced TF binding in the regulatory regions of miRNA genes.

Future work on dPORE-miRNA will include the integration of new promoter sets as soon as they become available. In addition, we plan to update the database in a next iteration to include the latest genome build (HG19) and the newest available SNP mapping (dbSNP 132). We also plan to integrate further search interfaces to increase dPORE-miRNA's utility, e.g. enable comparisons between miRNA genes with regards to SNPs and TFs.

The database dPORE-miRNA, as presented here, complements existing repositories and represents an easy means to investigate the regulatory regions of specific miRNAs of interest for SNPs that potentially affect miRNA regulation and shows the specific effects on binding sites.
